# Evaluation of antibacterial activity of propolis on regenerative potential of necrotic immature permanent teeth in dogs

**DOI:** 10.1186/s12903-019-0835-0

**Published:** 2019-08-06

**Authors:** M. M. El-Tayeb, A. M. Abu-Seida, S. H. El Ashry, S. A. El-Hady

**Affiliations:** 1 Department of Endodontics, Faculty of Dentistry, Ain Shams University, Cairo, Egypt; 2 Department of Surgery, Anesthesiology & Radiology, Faculty of Veterinary Medicine, Cairo University, Giza Square, Giza, PO: 12211 Egypt; 3 Department of Microbiology & Immunology, Faculty of Medicine, Ain Shams University, Cairo, Egypt

**Keywords:** Apical closure, Dentin thickness, Propolis, regenerative endodontic, Root length, Triple antibiotic paste

## Abstract

**Background:**

This study evaluated the antibacterial efficiency and ability of propolis to promote regeneration of immature permanent non-vital dogs’ teeth.

**Methods:**

Ninety six immature permanent premolars teeth in 6 mongrel dogs were divided randomly into: experimental teeth (*N* = 72) and control teeth (*N* = 24). Periapical pathosis was induced in all experimental and positive control teeth. Experimental teeth were classified according to the used intra-canal medication into: group I (*N* = 36), propolis paste was used and group II (N = 36), triple antibiotic paste (TAP) was used. Bacteriologic samplings were collected before and after exposure to intra-canal medicaments. After the disinfection period (3 weeks), revascularization was induced in all experimental teeth. Each group was subdivided according to the root canal orifice plug into: subgroup A (*N* = 18), propolis paste was used and subgroup B (N = 18), mineral trioxide aggregates (MTA) was used. Each subgroup was further subdivided according to the evaluation period into 3 subdivisions (6 teeth each): subdivision 1; after 2 weeks, subdivision 2; after one month and subdivision 3; after 2 months. Positive control group had 12 teeth with induced untreated periapical pathosis. Negative control group had 12 untouched sound teeth. All teeth were evaluated with radiography and histology. The bacteriologic and radiographic data were analyzed using repeated measures ANOVA and post-hoc Tukey tests. The histologic data were analyzed using Kruskal-Wallis test, Mann-Whitney U test with Bonferroni’s adjustment and Chi-square test. The significance level was set at *P* ≤ .05.

**Results:**

There was no significant difference in the antibacterial effectiveness between TAP and propolis groups (*P* > .05). In all subdivisions, there was no significant difference between the experimental groups in terms of increase in root length and dentin thickness, decrease in apical closure, new hard tissue formation, vital tissue formation inside the pulp canal and apical closure scores (*P* > .05).

**Conclusion:**

Propolis can be comparable with TAP as a disinfection treatment option in regenerative endodontic. As a root canal orifice plug after revascularization of necrotic immature permanent teeth in dogs, propolis induces a progressive increase in root length and dentin thickness and a decrease in apical diameter similar to those of MTA.

## Background

Propolis is a natural substance, containing about 55% resinous compounds and balsam, 30% beeswax, 10% ethereal and aromatic oils, and 5% bee pollen. This composition depends upon the type of plants available to the bees. Therefore, propolis changes in odor, color and probably therapeutic properties according to the season and source [1].

Due to the diversity of its therapeutic and biological properties, several recent studies have been carried out on propolis medical and dental applications [2, 3]. In dentistry, propolis has been used for the treatment of periodontitis, root canal disinfection, pulp capping and as a subsidiary treatment for gingivitis and plaque without any recorded allergic reactions [3, 4].

Propolis has antimicrobial and anti-inflammatory properties, so it could be applied as an effective intra-canal irrigant and intra-canal medicament [5–7]. Previous in vitro studies concluded that propolis has a good antibacterial activity against *E. faecalis* in the root canals, suggesting that it may be used as an alternative intra-canal medicament [6].

Successful endodontic therapy is based primary upon removal of as many bacteria as possible from the root canal and creation of unsuitable environment for the remaining microorganisms. In this regard, several intra-canal medicaments have been applied but no single agent has been found to be completely effective. Therefore, topical application of TAP consisting of a mixture of ciprofloxacin, metronidazole and minocycline or doxycycline is commonly used for sterilization of infected root canal [8]. Due to the disadvantages of TAP, search for an ideal new intra-canal antimicrobial is continuing. Therefore, propolis, as a natural product with useful biological activities and no reported complications, was selected in the present study.

Recently, regenerative endodontic treatment is advised for treatment of non-vital immature permanent teeth [9–11]. Revascularization of the necrotic dental pulp is the most commonly applied regenerative endodontic treatment [12, 13].

The hypothesis of this study was that propolis may play a role in the regenerative endodontic due to its antimicrobial, anti-inflammatory, analgesic, antioxidant, and immune stimulant activities. Therefore, this study evaluated the antibacterial efficiency of propolis and its ability to promote the regeneration as a root canal orifice plug after revascularization of immature non-vital dogs’ teeth.

## Methods

### Animals

The present study was approved by the Institutional Animal Care and Use Committee at Faculty of Dentistry, Ain Shams University, Egypt (No: FDASU-REC-16-2012). The authors followed up all institutional and international guidelines for animal care and use during this study. The Animal Research: Reporting in Vivo Experiments guidelines (ARRIVE) were also followed up. Four premolars were used in each quadrant of six apparently healthy immature mongrel stray dogs. These animals were obtained commercially from Al-Fahad Trading Company for Animals (Abu-Rawash, Giza, Egypt). The animals were of both sexes and their weight and age ranged between 10 and 15 kg (mean12.5 ± 0.5) and 6–9 months (mean 7.5 ± 0.5), respectively. Each dog was subjected to a full physical examination by an expert veterinarian to exclude any diseased dog. The animals were kept in the animal house at Faculty of Veterinary Medicine, Cairo University under proper conditions of nutrition, ventilation, clean environment and 12 h light/dark cycle. They were kept on separate kennels (1.5 m × 2.5 m × 3 m) and acclimatized to housing and diet for two weeks before the experiment. The dogs were given two meals per day (Soft food and milk) and fresh water ad libitum.

### Classification of samples

The samples (96 teeth) were divided using simple randomization into: experimental teeth (*N* = 72) and control teeth (*N* = 24). The experimental teeth were classified according to the used intra-canal medications into: group I (*N* = 36 teeth); the root canals were medicated with pure propolis paste and group II (N = 36 teeth); the root canals were medicated with TAP.

Each group was subdivided according to the used regenerative material into: subgroup (A); the root canal orifice was plugged with propolis paste (*N* = 18 teeth) and subgroup (B); the root canal orifice was plugged with MTA (N = 18 teeth). All experimental and control groups and subgroups were represented in each dog. The first mandibular premolars in each dog were used as positive control and the fourth maxillary premolars were used as negative control. The other three premolars in each quadrant represented randomly one experimental subgroup.

Each subgroup was further subdivided according to the evaluation period into 3 subdivisions (6 teeth each): subdivision (1); after 2 weeks, subdivision (2); after one month and subdivision (3); after 2 months.

The control teeth (*N* = 24 teeth) were classified into: positive control group which represented 12 teeth with induced periapical infections without any treatment modalities and negative control group which represented 12 sound teeth left untouched for normal maturation.

### Induction of infection

General anesthesia was administrated after fasting the dogs for 12 h. All dogs were pre-medicated with subcutaneous injection of Atropine sulphate (Atropine sulphate®, ADWIA Co., Egypt), 0.05 mg/kg weight and Xylazine HCl (Xylaject 2%® ADWIA Co., Egypt), 1.1 mg/kg body weight given intravenously. The anesthesia was induced with Ketamine HCl (Keiran®, EIMC Pharmacuticals Co., Egypt) at a dose of 5 mg/kg body weight via a 20-gauge IV cannula fixed in the cephalic vein. Anesthesia was maintained by 25 mg/kg incremental doses of Thiopental sodium 2.5% solution (Thiopental sodium®, EIPICO, Egypt). Radiographic examination was performed for all teeth to confirm incomplete root formation and to establish a base line working length for further comparison.

Endodontic access cavity was performed using size #2 diamond stone in all experimental and positive control teeth to exposing the pulp chamber. A sterile file size #40 was used to disrupt the pulp tissue. Supra gingival plaque from the dog’s teeth was mixed with sterile saline solution; sterile sponge was soaked in the plaque suspension and then inserted into the pulp chamber. Dogs were monitored radiographically after four weeks for evidence of development of periapical pathosis. Dogs were fed soft diet and given Carprofen (Rimadyl tab®, Pfizer Co., USA) daily at a dose of 4.4 mg/kg body weight as a pain killer during this period. The dogs were monitored daily for pain.

### Treatment modalities

#### Phase I: antibacterial effectiveness

Under the same general anesthetic regimen, complete aseptic conditions and cotton roll isolation, the previously infected experimental teeth were re-entered. The soaked cotton was removed and each root canal was filled with sterile saline solution as a transport fluid. A sterile paper point #30 was placed in the root canal as close to the working length as possible, allowed to saturate, and transferred into tubes containing one mL of 0.9% sterile saline solution. Before placing the paper point into the tubes, the mouth of each tube was heated on the flame to prevent contamination.

Each sample was carefully homogenized by being vortexed for 30s. Serial 10-fold dilutions (1:10, 1:100 and 1:1000) were done in saline solution. Then 0.1 mL from each dilution was smeared to be inoculated on surface of the plate media (BHI agar plates), incubated at 37 °C for 48 h, and colony-forming units (CFU) per 1 mL were enumerated. After 48 h, Petri-dishes were examined for bacterial growth (base line). Each plate was divided into 4 quadrants and dotted colonies were marked by a marker pen.

According to the group, either propolis paste or TAP paste was applied as intra-canal medicament.

For preparation of intra-canal propolis paste, Egyptian propolis was collected from El Monofia province. Three-hundred grams of frozen propolis were ground and dissolved in 300 mL of ethanol 96% at 37 °C to obtain 100% (w/v) extract. The mixture was poured into a bottle and incubated for 2 weeks at 30 °C. After incubation, the supernatant mixture was filtered twice with Whatman no. 4 and 1 filter paper. The filtered mixture was concentrated at 30 °C for 6 h (1500 rpm). The final extraction of propolis obtained a density of 150 mg/mL. Glycerin drops (3–4 drops) were added to 150 mg of the final extract of propolis until a creamy paste was obtained. For preparation of propolis orifice plug, the same steps were applied but the final extract of propolis (150 mg) was manipulated with 1–2 drops of glycerin until a thick paste, applicable for orifice plugging, was obtained.

Triple Antibiotic Paste consisting of Metronidazole 500 mg tablet, Ciprofloxacin 250 mg tablet and Doxycycline 100 mg capsule was prepared according Tawfik et al. [14]. Briefly, the Doxycycline capsule content, a tablet of Metronidazole and a tablet of Ciprofloxacin were crushed and ground into homogenous powder in a sterile mortar using a pestle. Saline drops (6–8 drops) were added and mixed until a creamy paste was obtained. Injection of 1–1.5 mL of the prepared pastes into each canal was carried out to fill the canals using a lentulo spiral. The access cavity was sealed using sterile cotton pellet and temporary restoration, Coltosol® F (Coltene Whaledent, Switzerland). Samples were left for 3 weeks.

Under the same aseptic conditions and anesthesia, the temporary restoration, cotton pellet, and the paste were removed by copious irrigation with 10 mL sterile saline solution. The root canals were dried with sterile paper points. Then post exposure to intra-canal medicaments bacteriologic sampling was performed by the previously described method. After bacteriologic sampling, the root canals were re-irrigated using 10 mL of NaOCl 2.25% solution then 10 mL of sterile saline solution. The root canals were dried with sterile paper points for regeneration protocol.

For bacterial count, visible colonies produced before and after intra-canal medicaments were counted in every plate. The number of colonies/plate was multiplied by the corresponding dilution factor and by 10 to determine the total colony forming units (CFU) per mL of sample. Antibacterial effectiveness of the different intra-canal medicaments was assessed by determining the percentage of reduction in colony counts (%RCC) before and after application of the intra-canal medicaments.

The percentage of change was calculated as:

$$ \frac{\mathrm{CFU}\ \left(\mathrm{Base}\ \mathrm{line}\right)-\mathrm{CFU}\ \left(3\ \mathrm{weeks}\right)}{\mathrm{CFU}\ \left(\mathrm{Base}\ \mathrm{line}\right)}\times 100 $$

#### Phase II: regeneration protocols

A sterile hand file size # 30 was inserted past to the canals terminus for induction of bleeding that filled the canal space just below the CEJ. After clotting of the blood, the experimental teeth were divided randomly and either propolis (subgroup A) or MTA (subgroup B) orifice plug was used to seal the canal orifice. The plugs were covered by a moist cotton pellet and the access cavity was closed with glass ionomer filling (Medifill®, Promedica, Germany).

#### Radiographic evaluation

Periapical radiographs were taken after 4 weeks of induction of periapical pathosis and at 2, 4 and 8 weeks after revascularization to verify healing of the periapical pathosis. TurboReg plug-in (TurboReg®, Biomedical Imaging Group, Swiss Federal Institute of Technology, Lausanne VD, Switzerland) was used to transform the non-standardized pre-operative and post-operative radiographs to standardized images. Both increase in root length and thickness and decrease in apical diameter were evaluated according to Tawfik et al. [14].

#### Histopathologic evaluation

According to the post-treatment evaluation period, the animals were sacrificed by overdose of Thiopental sodium (20 mL of 5% solution given intravenously at once). The teeth and surrounding bone were fixed, prepared and stained with hematoxylin and eosin for histopathologic assessment. Quantitative histologic evaluation was performed according to Tawfik et al. [14]. This evaluation included; presence or absence of both new hard tissue on internal dentinal walls and vital tissues inside the pulp canal as well as apical closure.

Qualitative histologic analysis was carried out. Histologic findings of hard structure included; dentin (presence of dentinal tubules), cementum (adherence to dentin and presence of cementocyte-like cells), bone (presence of Haversian canals with uniformly distributed osteocyte-like cells) and periodontal ligament (bridging of Sharpey’s fibers between cementum and bone).

### Statistical analysis

IBM (IBM®, NY, USA) SPSS® Statistics Version 20 for Windows (SPSS, Inc., IBM Company, USA) was applied for statistical analysis. Numerical data were presented as mean and standard deviation (SD) values. Data were explored for normality using Kolmogorov-Smirnov and Shapiro-Wilk tests. A logarithmic transformation (Log_10_ transformation) of each CFU count was performed because of the high range of bacterial counts. Histological scores were tested as non-parametric data while radiographic data showed parametric distribution.

As regards Log_10_ CFU data, % increase in root canal dentin thickness and % decrease in apical diameter; repeated measures ANOVA test was used to compare between the different groups as well as to compare between the different subdivisions. Tukey’s post-hoc test was used for pair-wise comparisons between the groups when ANOVA test was significant. Kruskal-Wallis test was applied to compare between histologic scores of the different groups and the different subdivisions. Mann-Whitney U test with Bonferroni’s adjustment was used for pair-wise comparisons between the groups when Kruskal-Wallis test is significant. Prevalence of apical closure data was presented as frequencies (n) and percentages (%). Chi-square (*x*^*2*^) test was performed to compare between the groups and subdivisions. The significance level was set at *P* ≤ .05.

## Results

Clinically all dogs ate and drank well. No signs of pain and no allergic reactions were reported in any dog during this study.

### Phase I (antimicrobial effectiveness)

At the base line and after 3 weeks, positive control group showed the highest mean log_10_ CFU. There was a significant increase in mean log_10_ CFU of bacterial counts after 3 weeks in the control positive group (*P* < .001). As regards percentage of change in bacterial counts, there was no significant difference between TAP and propolis groups (*P* > .05). Both groups showed a decrease in percentage of reduction in log_10_ CFU. Negative control group showed no change with a recorded mean value of .00 ± .00% (Table 1).

**Table 1:** The mean log_10_, standard deviation (SD) values and results of repeated measures ANOVA and Tukey’s tests for comparison between log_10_ CFU of bacterial counts in all groups at different time periods

Time	TAP group	Propolis group	Positive control	Negative control	*P*-value
	Mean log_10_ ± SD	Mean log_10_ ± SD	Mean log_10_ ± SD	Mean log_10_ ± SD	
Base line	3.65 ^Ab^ ± 0.33	3.88 ^Ab^ ± 0.15	4.00 ^Aa^ ± 0.02	0.00 ^Ac^ ± 0.00	<.001*
3 weeks	2.63 ^Bb^ ± 0.13	2.99 ^Bb^ ± 0.09	4.84 ^Ba^ ± 0.40	0.00 ^Ac^ ± 0.00	<.001*
*P*-value	.015*	.002*	.003*	Not computed	

*: Significant at *P* ≤ .05, Different small superscripts in the same row are statistically significantly different. Different capital superscripts in the same column are statistically significantly different. *TAP:* Triple antibiotic paste

### Phase II (regeneration)

#### Radiographic findings

**Fig. 1:**
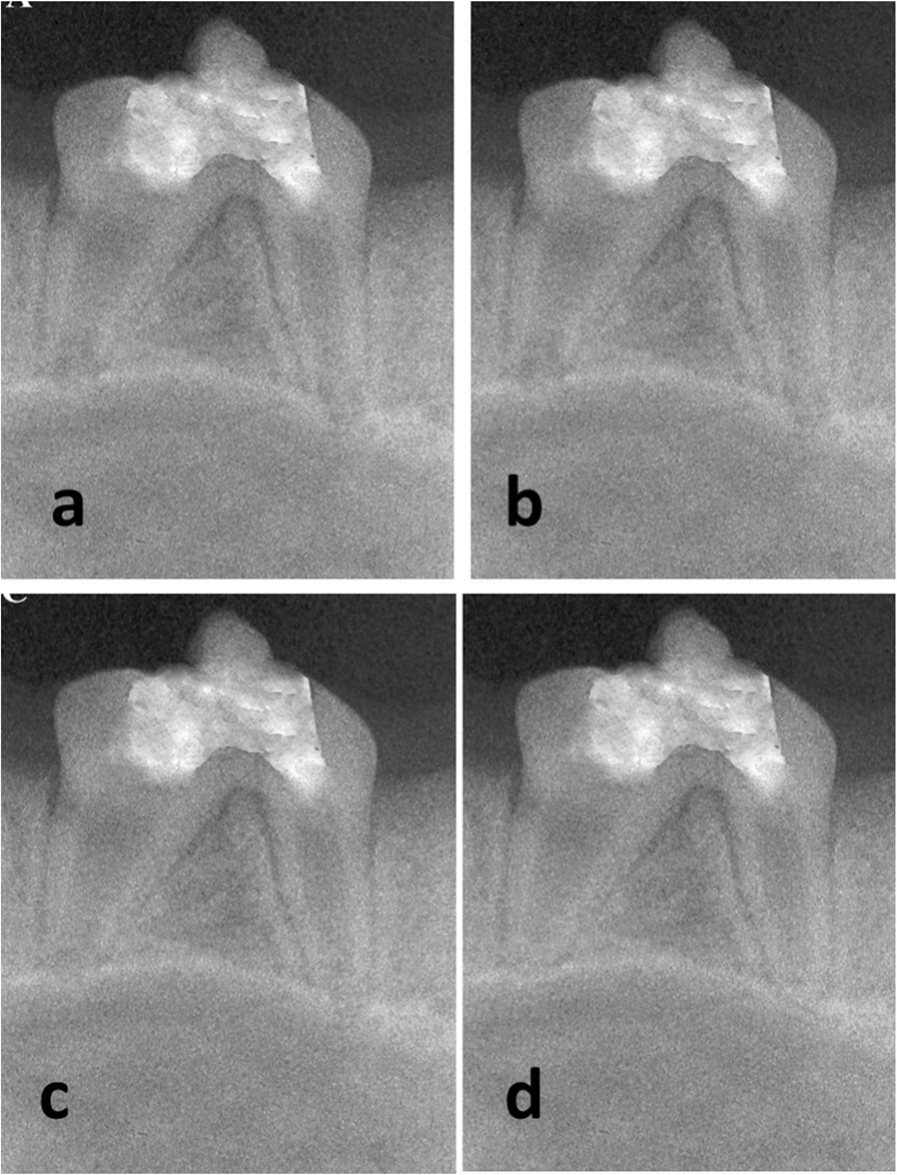
Representative radiographs of subgroup (A); Propolis over empty canal. (**a**): Preoperative radiograph. Postoperative radiographs two weeks (**b**), one month (**c**) and two months (**d**) after revascularization protocol

**Fig. 2:**
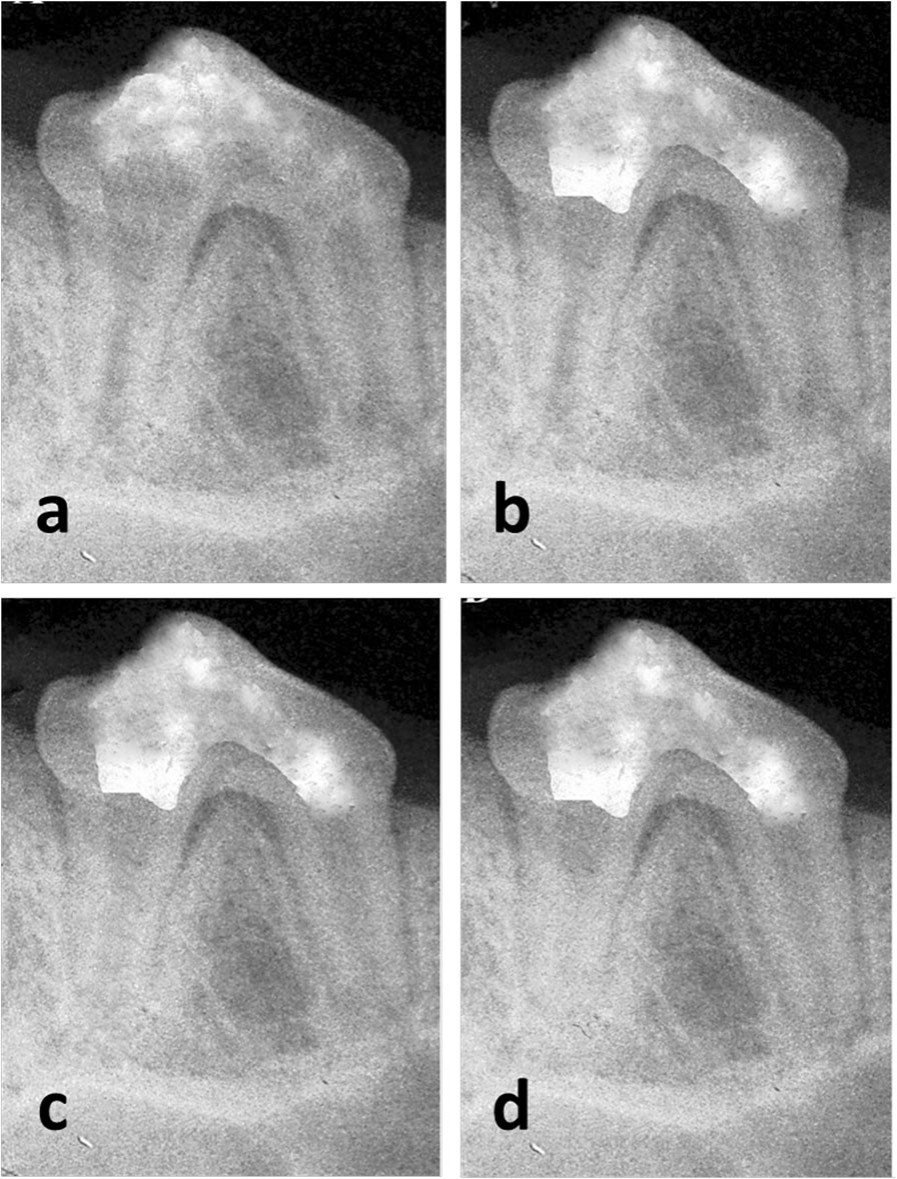
Representative radiographs of subgroup (B); MTA over empty canal. (**a**) Preoperative radiograph. Postoperative radiographs two weeks (**b**), one month (**c**) and two months (**d**) after revascularization protocol

The experimental groups showed a progressive increase in root length and dentin thickness and a decrease in apical diameter with no significant difference (*P* > .05) at all evaluation periods (Tables 2, 3 and 4 and Figs. 1 and 2).

**Table 2:** The mean, standard deviation (SD) values and results of repeated measures ANOVA and Tukey’s tests for percentage of increase in root length of different groups, subgroups and subdivisions

Time	Intra-canal propolis paste		Intra-canal TAP paste		Positive control	Negative control	*P*-value
	Propolis plug	MTA plug	Propolis plug	MTA plug			
	Mean ± SD	Mean ± SD	Mean ± SD	Mean ± SD	Mean ± SD	Mean ± SD	
2 W	4.9 ± 1.2	5.4 ± 1.4	5.1 ± 1	5.3 ± 1.3	0.00 ± 0.00	5.7 ± 1.3	.061
1 M	13.9 ^Aa^ ± 1.8	14.6 ^Aa^ ± 1.5	14 ^Aa^ ± 1.6	14^Aa^ ± 1.6	0.00^Bb^ ± 0.00	14.8^Aa^ ± 1.8	<.001*
2 M	16.3^Bb^ ± 1.3	17.1 ^Bb^ ± 1.6	16.5^Bb^ ± 1.4	16.9 ^Bb^ ± 2.3	0.00^Cc^ ± 0.00	17.7^Aa^ ± 1.5	<.001*
*P*-value	<.001*	<.001*	<.001*	<.001*	Not computed	<.001*	

*: Significant at *P* ≤ .05, Different small superscripts in the same row are statistically significantly different. Different capital superscripts in the same column are statistically significantly different. *TAP:* Triple antibiotic paste, *MTA:* Mineral trioxide aggregates

**Table 3:** The mean, standard deviation (SD) values and results of repeated measures ANOVA and Tukey’s tests for percentage of increase in root canal dentin thickness of different groups, subgroups and subdivisions

Time	Intra-canal propolis paste		Intra-canal TAP paste		Positive control	Negative control	*P*-value
	Propolis plug	MTA plug	Propolis plug	MTA plug			
	Mean ± SD	Mean ± SD	Mean ± SD	Mean ± SD	Mean ± SD	Mean ± SD	
2 W	3.87^B^ ± 0.54	4.60^B^ ± 0.61	3.95^B^ ± 0.53	4.72^B^ ± 0.60	0.00 ± 0.00	5.02^B^ ± 0.75	.060
1 M	6.20^Ba^ ± 0.74	7.83^Ba^ ± 0.86	6.35^Ba^ ± 1.20	7.06^Ba^ ± 2.10	0.00^b^ ± 0.00	8.40^Ba^ ± 1.12	<.001*
2 M	13.28^Ab^ ± 0.79	13.92^Ab^ ± 0.85	13.22^Ab^ ± 2.41	14.50^Ab^ ± 1.98	0.00^c^ ± 0.00	17.62^Aa^ ± 0.54	<.001*
*P*-value	<.001*	<.001*	<.001*	<.001*	Not computed	<.001*	

*: Significant at *P* ≤ .05, Different small superscripts in the same row are statistically significantly different. Different capital superscripts in the same column are statistically significantly different. *TAP*: Triple antibiotic paste, *MTA*: Mineral trioxide aggregates

**Table 4:** The mean, standard deviation (SD) values and results of repeated measures ANOVA and Tukey’s tests for percentage of decrease in apical diameter of different groups, subgroups and subdivisions

Time	Intra-canal propolis paste		Intra-canal TAP paste		Positive control	Negative control	*P*-value
	Propolis plug	MTA plug	Propolis plug	MTA plug			
	Mean ± SD	Mean ± SD	Mean ± SD	Mean ± SD	Mean ± SD	Mean ± SD	
2 W	3.05^B^ ± 0.97	3.37^B^ ± 0.74	3.12^B^ ± 0.74	3.55^B^ ± 0.61	0.00 ± 0.00	4.00^B^ ± 1.15	.053
1 M	6.23^Ba^ ± 1.48	7.45 ^Ba^ ± 1.09	5.24^Ba^ ± 1.04	7.99^Ba^ ± 2.12	0.00^b^ ± 0.00	8.23^Ba^ ± 1.09	<.001*
2 M	30.50^Ab^ ± 1.84	31.77^Ab^ ± 1.04	32.48^Ab^ ± 4.20	34.00^Ab^ ± 5.59	0.00^c^ ± 0.00	46.85^Aa^ ± 1.03	<.001*
*P*-value	<.001*	<.001*	<.001*	<.001*	Not computed	<.001*	

*: Significant at *P* ≤ .05, Different small superscripts in the same row are statistically significantly different. Different capital superscripts in the same column are statistically significantly different. *TAP*: Triple antibiotic paste, *MTA*: Mineral trioxide aggregates

#### Increase in root length

After 2 weeks; there was no significant difference in terms of increase in root length between all groups (*P* = .061).

After 1 month, there was no significant difference in terms of increase in root length between the experimental and negative control groups (*P* > .05).

After 2 months; the negative control samples showed the highest increase in root length. There was no significant difference in terms of increase in root length between the experimental groups (*P* > .05). Positive control samples showed the lowest increase in root length after one month and 2 months periods.

As regards subdivisions, the two weeks period showed the lowest increase in root length. There was no significant difference between 2 weeks and 1 month periods in terms of increase in root length (*P* > .05). The two months period showed the highest increase in root length (Table 2).

#### Increase in dentin thickness

After 2 weeks; there was no significant difference in terms of increase in dentin thickness between all groups (*P* = .060).

After 1 month; there was a significant difference in terms of increase in dentin thickness between the experimental groups and negative control group and positive control samples (*P* < .001). There was no significant difference between experimental and negative control groups (*P* > .05). Positive control samples showed the lowest increase in dentin thickness.

After 2 months; the negative control samples showed the highest increase in dentin thickness. There was no significant difference in terms of increase in dentin thickness between the experimental groups (*P* > .05). The experimental groups showed a significant lower increase in dentin thickness than that of negative control group (*P* < .001). Positive control group showed the lowest increase in dentin thickness.

As regards subdivisions, two weeks period showed the lowest increase in dentin thickness. There was no significant difference between 2 weeks and 1 month periods (*P* > .05). The two months period showed the highest increase in dentin thickness compared to that of other subdivisions (Table 3).

#### Decrease in apical diameter

After 2 weeks; there was no significant difference in terms of decrease in apical diameter between the groups (*P* = .053).

After 1 month; there was a significant difference in terms of decrease in apical diameter between the groups (*P* < .001). There was no significant difference in terms of decrease in apical diameter between the experimental groups and negative control group (*P* > .05). The lowest percentage of decease in apical diameter was recorded in the positive control group.

After 2 months; there was a significant difference in terms of decrease in apical diameter between the groups (*P* < .001). Negative control group showed the highest decrease in apical diameter. There was no significant difference in terms of decrease in apical diameter between the experimental groups (*P* > .05). The experimental groups showed a significant lower decrease in apical diameter than that of negative control samples (*P* < .001). Positive control group showed no change in apical diameter after one month and two months periods.

Regarding subdivisions, two weeks period showed the most significant lowest decrease in apical diameter (*P* < .001). There was no significant difference in terms of decrease in apical diameter between 2 weeks and 1 month periods (*P* > .05). Two months period showed the highest decrease in apical diameter compared with that of other subdivisions. There was no decrease in apical diameter through all subdivisions in positive control group as shown in Table 4.

#### Histopathologic findings

Regarding the histopathologic findings, there was no significant difference in terms of hard tissue formation, vital tissues formation inside the pulp space and apical closure between the experimental groups at all evaluation periods (*P* > .05).

#### Findings of the quantitative analysis

##### Hard tissue formation scores

After 2 weeks, the score of hard tissue formation showed no significant difference between the experimental groups (*P* = .061).

After 1 month, there was no significant difference in terms of hard tissue formation between the experimental groups and negative control group (*P* > .05). Positive control group showed the lowest hard tissue formation score (Table 5).

**Table 5:** The mean, standard deviation (SD) values and results of Kruskal-Wallis and Mann-Whitney U tests for comparison between hard tissue formation scores of different groups, subgroups and subdivisions

Time	Intra-canal propolis paste		Intra-canal TAP paste		Positive control	Negative control	*P*-value
	Propolis plug	MTA plug	Propolis plug	MTA plug			
	Mean ± SD	Mean ± SD	Mean ± SD	Mean ± SD	Mean ± SD	Mean ± SD	
2 W	0.50^B^ ± 0.23	0.67^B^ ± 0.42	0.60^B^ ± 0.41	0.63^B^ ± 0.28	0.00 ± 0.00	0.68^B^ ± 0.44	.061
1 M	0.67^Ba^ ± 0.52	0.83^Ba^ ± 0.41	0.65^Ba^ ± 0.34	0.77^Ba^ ± 0.40	0.00^b^ ± 0.00	1.00^Ba^ ± 0.00	<.001*
2 M	1.17^Ab^ ± 0.41	1.50^Ab^ ± 0.55	1.30^Ab^ ± 0.60	1.55^Ab^ ± 0.65	0.00^c^ ± 0.00	2.0^Aa^ ± 0.61	<.001*
*P*-value	.040*	.037*	.042*	.038*	1.000	<.001*	

*: Significant at *P* ≤ .05, Different small superscripts in the same row are statistically significantly different. Different capital superscripts in the same column are statistically significantly different. *TAP*: Triple antibiotic paste, *MTA*: Mineral trioxide aggregates

After 2 months, the negative control group showed the most significant highest hard tissue formation score. There was no significant difference in terms of hard tissue formation between the experimental groups (*P* > .05). The experimental and positive control groups showed a significant lower hard tissue formation score than that of negative control group (*P* < .001). Positive control group showed the lowest hard tissue formation score after one month and 2 months.

There was a significant difference in terms of hard tissue formation score between the experimental and negative control groups through all subdivisions (*P* < .05).

Pair-wise comparison between the subdivisions revealed no significant difference in terms of hard tissue formation score between 2 weeks and 1 month periods (*P* > .05). Two months subdivision showed a significant higher hard tissue formation score than that of other subdivisions (*P* < .05). Positive control group showed no significant difference between hard tissue formation scores at all subdivisions (*P* = 1.000) as shown in Table 5.

##### Vital tissues formation inside the pulp space

After 2 weeks, there was no significant difference in terms of vital tissue formation inside the pulp space between all groups (*P* = .053).

After 1 month; there was no significant difference in terms of vital tissue formation inside the pulp space between the experimental and negative control groups *(P* > .001). The experimental and negative control groups showed a significant higher vital tissue formation inside the pulp space than that of positive control group *(P* > .001).

After 2 months; the negative control group showed the significant highest vital tissue formation inside the pulp space. There was no significant difference in terms of vital tissue formation inside the pulp space between the experimental groups *(P* > .05). The experimental groups showed lower vital tissue formation inside the pulp space than that of negative control group. Positive control group showed no vital tissue score after one month and two months.

There was a significant difference in terms of vital tissue formation inside the pulp space between the experimental and negative control groups at all evaluation periods (*P* < .05) as shown in Table 6. There was no significant difference between 2 weeks and 1 month periods (*P* > .05). Two months period showed the most significant highest vital tissue formation inside the pulp space compared with that of 2 weeks and 1 month periods. There was no significant difference between vital tissue formation scores at all evaluation periods (*P* = 1.000) as shown in Table 6.

**Table 6:** The mean, standard deviation (SD) values and results of Kruskal-Wallis and Mann-Whitney U tests for comparison between vital tissue formation inside the pulp space of different groups, subgroups and subdivisions

Time	Intra-canal propolis paste		Intra-canal TAP paste		Positive control	Negative control	*P*-value
	Propolis plug	MTA plug	Propolis plug	MTA plug			
	Mean ± SD	Mean ± SD	Mean ± SD	Mean ± SD	Mean ± SD	Mean ± S D	
2 W	0.67 ^B^ ± 0.52	0.67 ^B^ ± 0.52	0.52 ^B^ ± 0.42	0.70 ^B^ ± 0.45	0.00 ± 0.00	0.67 ^B^ ± 0.52	.053
1 M	1.17 ^Ba^ ± 0.75	1.50 ^Ba^ ± 0.55	1.22^Ba^ ± 0.39	1.56^Ba^ ± 0.69	0.00^b^ ± 0.00	1.53 ^Ba^ ± 0.62	.001*
2 M	1.83 ^Ab^ ± 0.41	2.00 ^Ab^ ± 0.00	1.95 ^Ab^ ± 0.67	2.06 ^Ab^ ± 0.70	0.00^c^ ± 0.00	2.50^Aa^ ± 0.47	.010*
*P*-value	.002*	.003*	.035*	.016*	1.000	.006*	

*: Significant at *P* ≤ .05, Different small superscripts in the same row are statistically significantly different. Different capital superscripts in the same column are statistically significantly different. *TAP*: Triple antibiotic paste, *MTA*: Mineral trioxide aggregates

##### Apical closure

After 2 weeks; no group showed apical closure.

After 1 month; there was no significant difference in terms of apical closure between the groups (*P* = .172).

After 2 months; there was a significant difference between the groups (*P* = .010). Negative control group showed the highest prevalence of apical closure while positive control group had no apical closure. Experimental groups showed a low and equal prevalence of apical closure.

High prevalence of apical closure was found after 1 month in all experimental and negative groups. Further increase in prevalence of apical closure was observed after 2 months. In negative control group, there was a significant difference in terms of apical closure between the evaluation periods (*P* = .002) as shown in (Table 7).

**Table 7:** The frequencies (n), percentages (%) and results of Chi-square test for apical closure in different groups, subgroups and subdivisions

Time	Intra-canal propolis paste				Intra-canal TAP paste				Positive control		Negative control		*P*-value
	Propolis plug		MTA plug		Propolis plug		MTA plug						
	N	%	N	%	N	%	N	%	N	%	N	%	
2 W	0	0.0	0	0.0	0	0.0	0	0.0	0	0.0	0	0.0	Not computed
1 M	2	33.3	3	50.0	3	50.0	3	50.0	0	0.0	4	66.7	0.172
2 M	4	66.7	4	66.7	4	66.7	4	66.7	0	0.0	6	100.0	0.010*
*P*-value	0.050*		0.017*		0.048*		0.048*		Not computed		0.002*		

*: Significant at *P* ≤ 0.05. *TAP*: Triple antibiotic paste, *MTA*: Mineral trioxide aggregates

##### Findings of the qualitative analysis

**Fig. 3:**
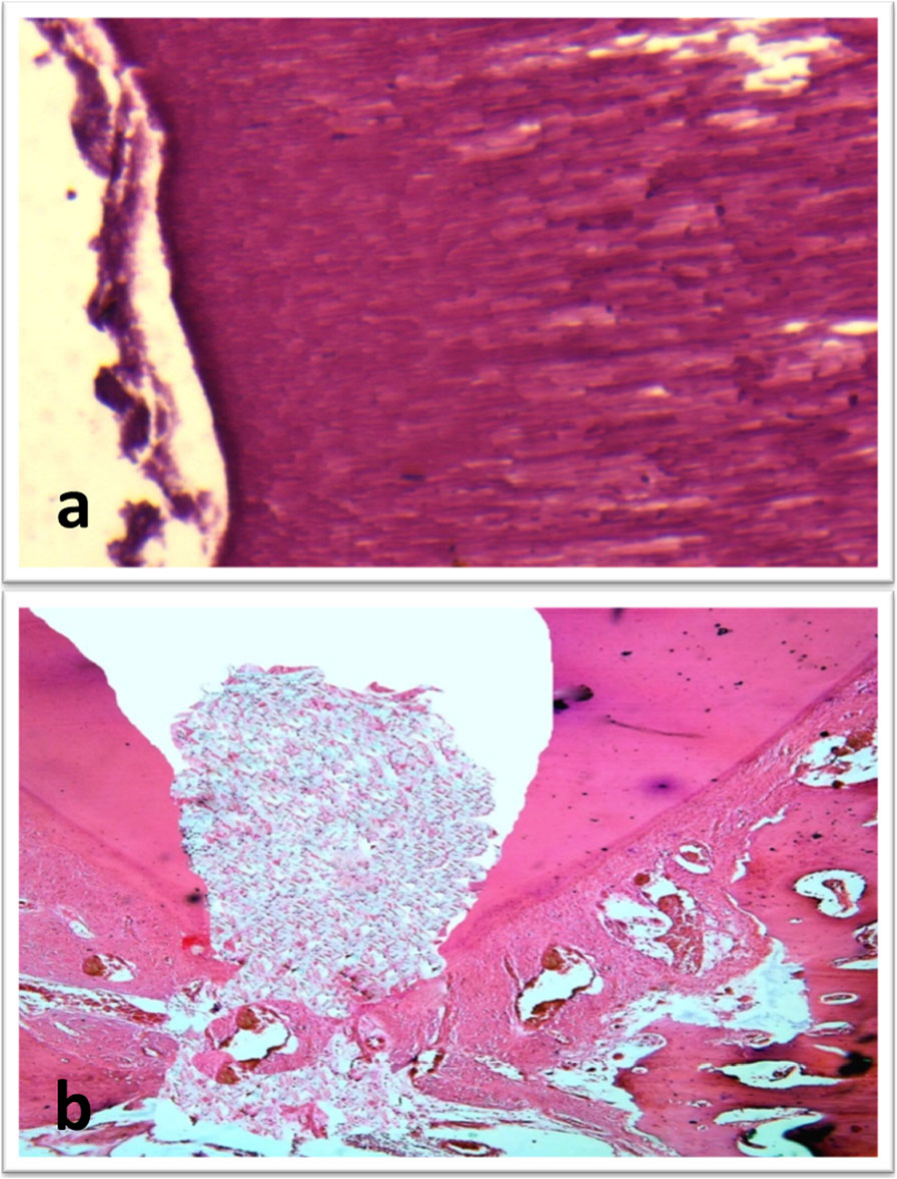
(**a**) Photomicrograph for a sample of subgroup A (propolis) showing formation of cementum like tissue on the inner aspect of the root dentin (H&E, X200). (**b**) Photomicrograph for a sample of subgroup A showing pulp like tissue with areas of osteoid like tissue inside the root canal and closure of the apex (H&E, X100)

In subgroup A (propolis), changes within the hard and soft tissues along the three evaluation periods were observed. Cementum like tissue was observed along the inner surface of root dentin resulting in an increase in the root thickness. Most of the teeth showed closure of the apical terminus of the root with cementum like tissue. Pulp like fibrous tissue devoid of odontoblastic layer with calcified islands of osteoid tissue was seen (Fig. 3).

**Fig. 4:**
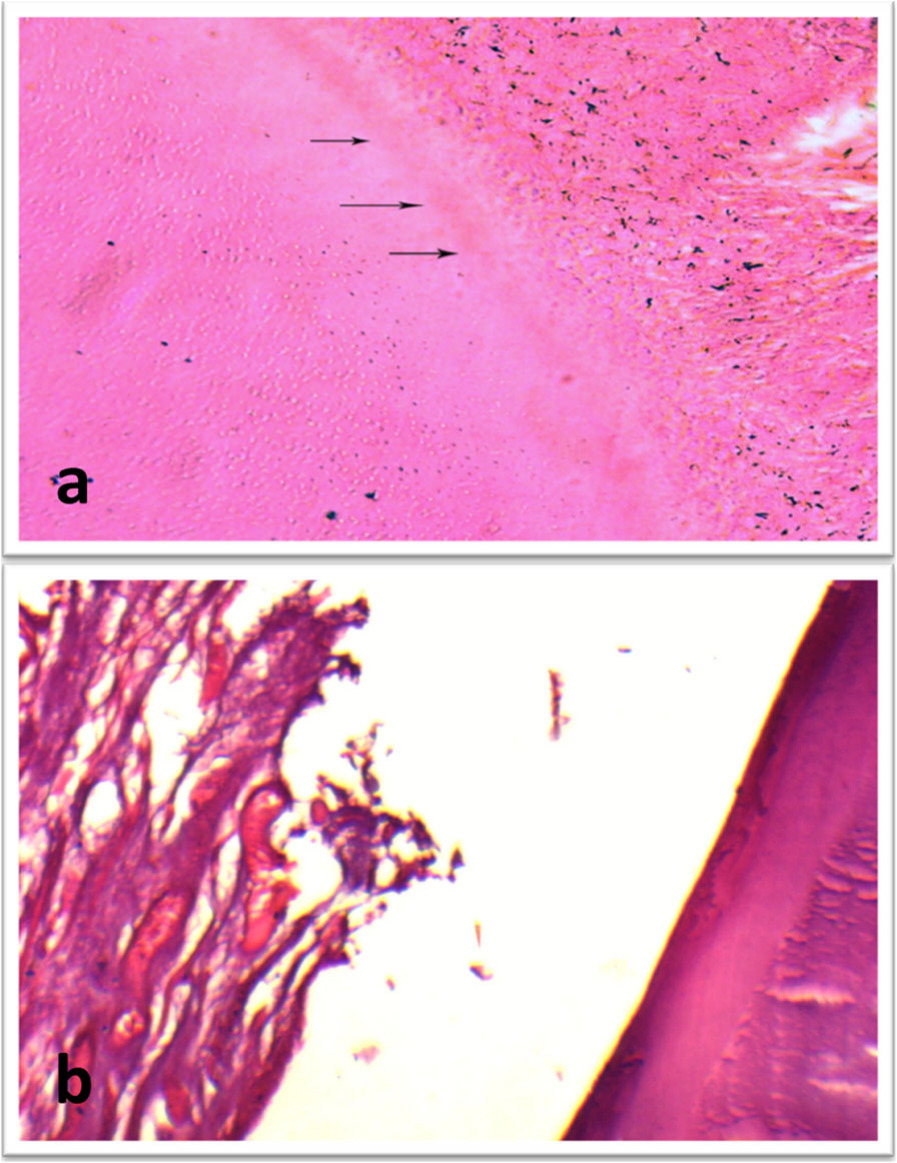
(**a**) Photomicrograph for a sample of subgroup B (MTA) at 2 month showing formation of new layer of dentin like tissue (arrows) on the inner root wall (H&E, X200). (**b**) Higher magnification of a sample of subgroup A (propolis) showing fibrous connective tissue with islands of cementum like tissue inside the root canal space (H&E, X200)

**Fig. 5:**
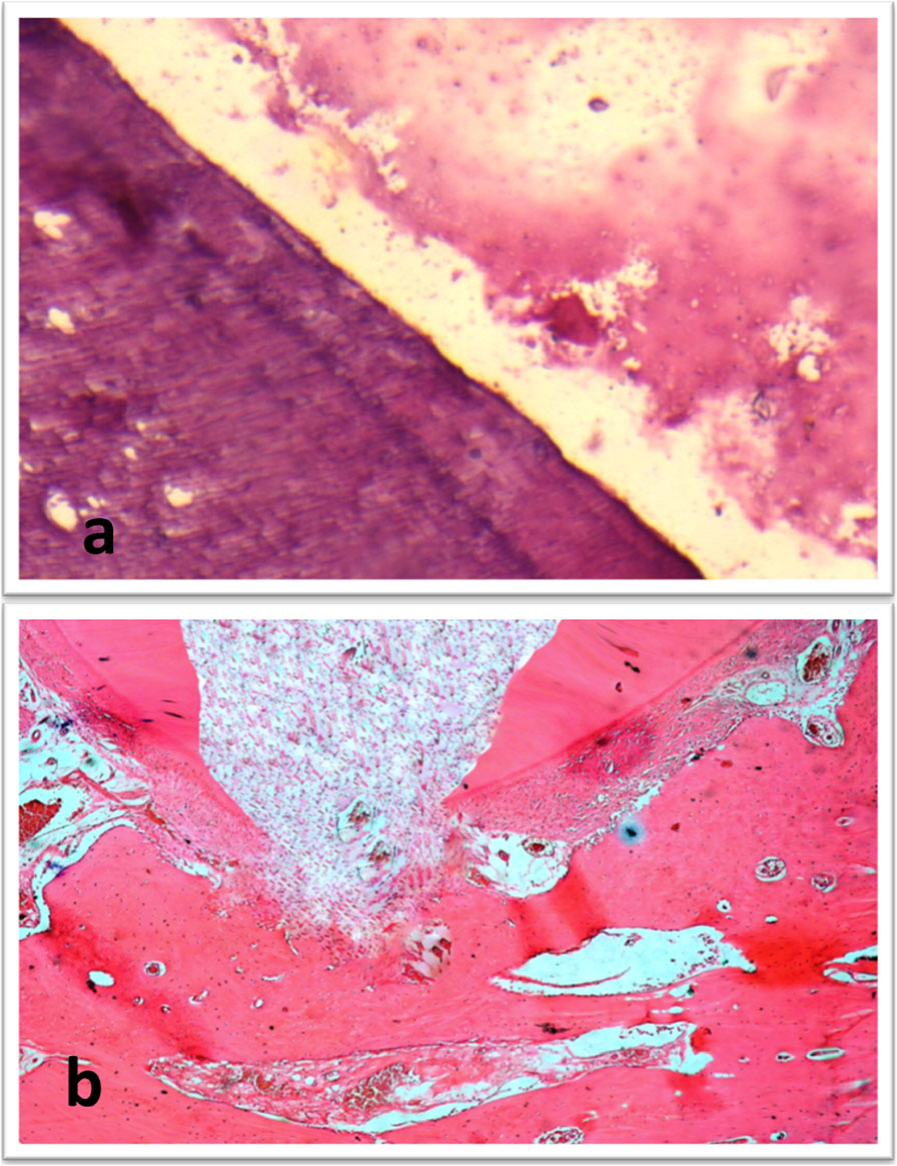
(**a**) Photomicrograph for a sample of subgroup B (MTA) at 1 month showing dentin like tissue formation (H&E, X200). (**b**) Photomicrograph for a sample of subgroup B showing pulp like tissue with areas of osteoid like tissue inside the root canal and closure of the apex (H&E, X100)

In subgroup B (MTA), changes within the hard and soft tissues along the three evaluation periods were observed. Teeth showed formation of cementum like tissue along the inner aspect of root dentin resulting in an increase in dentin thickness. Some teeth showed a newly formed layer of dentin along the inner aspect of the root leading to an increase in the root thickness (Fig. 4). Most of the teeth showed closure of the apical terminus of the root with cementum formation; however closure of the apical terminus with dentin formation was demonstrated in some teeth (Fig. 5). Pulp like fibrous connective tissue with no odontoblastic layer was seen in most teeth. The teeth with dentin formation were accompanied with the presence of pulp like tissue with odontoblastic layer.

## Discussion

A complete elimination of necrotic tissues and infection from the root canal is essential for a successful endodontic therapy [15]. Moreover, treatment of immature necrotic teeth constitutes a great challenge due to weakness of dentinal walls and difficult apical sealing [16].

In last few years, regenerative endodontic has gained much attention as an alternative biological treatment of immature permanent necrotic teeth because it allows further root maturation [17, 18]. Revascularization through induction of bleeding inside the dental pulp is a simple technique for regenerative endodontic [12, 13].

Due to its ant-inflammatory, antibacterial and biocompatibility features, propolis had been studied for several medical uses. Propolis has been used in dentistry for treatment of aphthous ulcers, periodontitis, *Candida albicans* and root canal disinfection due to its advantages; it does not stain the tooth crown, inhibits the formation of plaque, is preserved in the root canal, and enhances the bone regeneration [19–22].

Accordingly, the aims of this study were to assess both antibacterial efficiency of propolis during management of infected root canal and the ability to promote regeneration after revascularization for management of immature non-vital dogs’ teeth.

The dog was selected as an experimental animal for this biological experiment due to his similar apical repair compared with that of humans but in a short time (average one sixth of human) due to high growth rate [23]. Moreover, the selected dogs aged 6–9 months which are suitable for assessment of the immature teeth and withstanding of general anesthesia [14]. Dogs^,^ premolars were selected for this study due to their suitable-sized root canals for different endodontic manipulations and they are easily accessible.

Access cavities were performed in the selected teeth and left open for four weeks in order to induce periapical infection, for simulation of clinical cases. The periapical pathosis was confirmed by radiographic examination as mentioned before [13].

Failure of using rubber dam for isolation was attributed to the morphology of dogs^,^ premolars that makes fixation of the rubber dam clamp to the tooth difficult. Therefore, isolation was achieved by atropine injection to decrease salivation during surgery and cotton rolls.

In our study, no instrumentation was performed due to thin root dentinal walls of immature teeth. Similar finding was recorded before [24]. Therefore, disinfection of immature permanent teeth with necrotic pulp is entirely depended on the chemical action of intra-canal agents. However this is also another challenge because these agents may affect the viability of regeneration cells [25].

In the present study, the antimicrobial agents were kept inside the dental roots for 3 weeks which is a sufficient time for canal disinfection as mentioned before [8].

Due to the complexity of root canal infection, single antimicrobial agent is not sufficient for effective disinfection [26]. Thus mixed antimicrobials are more likely to use during endodontic therapy. Although triple antibiotic paste is one of the most commonly used antimicrobial combinations in endodontics, it has some drawbacks such as staining of the teeth [8]. Therefore, propolis was used in the present study as an alternative intra-canal medication in revascularization due to its natural origin and its ability to maintain PDL cell viability when used as a storage medium of avulsed teeth [27]. So these were the hypotheses for assessment of the regenerative potential of propolis in the current study.

In the present study, ethanol extract of propolis was used because it promotes the regeneration of bone and hard tissue bridge formation. Additionally, using of a scaffold is an essential factor for a successful regenerative endodontic because it supports cell proliferation, organization, differentiation and vascularization. In the present study, the blood clot was used as a scaffold because it is an easy, efficient and biological process. On the other hand, using other natural scaffolds in previous studies resulted in little success with several drawbacks [14, 17, 28].

Regarding the antimicrobial action of both propolis and TAP, there was no significant difference between them. The antimicrobial activity of propolis could be attributed to functional and structural damages of the microbial cell wall, inhibition of bacterial RNA-polymeras and immunomodulatory, anti-oxidative, and healing effects [19, 22].

Both radiographic and histologic examinations are necessary for assessment of healing of immature necrotic teeth [12–14]. The present radiographic findings were generally consistent with the histologic findings. These findings are in agreement with those reported before [9, 12, 14, 29]. However, Wang et al. [11] concluded that radiographic findings were not accurate regarding actual increase in length or thickness of the dental root due to different angulations and image resolution. That was not the case in our study due to radiographic standardization using Image-J with Turbo Reg plug in.

There was no significant difference between propolis and MTA subgroups regarding the change in root length and thickness due to the control of infection that was confirmed by the bacteriologic examination and the induction of blood clot in all subgroups [9].

Regarding new hard tissue formation, there was no significant difference between propolis and MTA subgroups along the three evaluation periods. The newly deposited hard tissue resembled cementum and had cementocyte-like cells. These results are in accordance with previous studies [18, 30]. Regarding the tissue in-growth, the nature of the regenerated tissue was pulp like fibrous connective tissue with calcified bony islands as well as cementum and without odontoblastic layer. These findings are in agreement with the findings of previous studies [17, 30]. On contrary, Tawfik et al. [14] found a newly formed tissue resembles periodontal tissue.

An interesting finding of this study is that some specimens from the propolis group showed pulp like tissue with the presence of odontoblastic layer. These findings could be attributed to the abundance amount of multi potent dental pulp stem cells in immature teeth, stem cells of the apical papilla, or remnants surviving pulp cells at the apical end of the root canal. These cells may proliferate in the blood clot and differentiate into odontoblasts under the influence of cells of Hertwig’s epithelial root sheath, which are resistant to destruction [31]. The ability of propolis to regenerate pulp is in accordance with findings of other researches which reported tubular dentin formation after pulp capping with propolis [3, 32].

The increase in root thickness by cementum deposition on the inner dentinal wall and the presence of cementum and bone inside the root canal could be due to delivering of mesenchymal stem cells from the bone and periodontal ligament into the root canal during instrumentation for induction of bleeding. Another theory is the blood clot itself which is rich in growth factors playing a crucial role in regeneration [33].

Apical closure is the last stage of root maturation, no significant difference was found between propolis and MTA subgroups regarding apical closure. Similarly, apical closure was reported after revascularization of dogs^,^ teeth with apical periodontitis [10].

General speaking, both radiographic and histologic findings showed no significant difference between both propolis and MTA subgroups at all evaluation periods, therefore propolis can be considered a substitute of MTA as a root canal orifice plug after revascularization.

Although toxicity data for propolis are scarce, few case reports of allergic reactions have been recorded after oral administration of propolis [34]. In our study, no allergic reactions were recorded. This could be attributed to application of propolis as intra-canal medicament and root canal orifice plug.

Long follow up period is recommended for monitoring of the regeneration process of the necrotic permanent immature teeth. Also, further studies are recommended for investigation of efficacy of propolis as an intra-canal medication in infected mature teeth.

## Conclusions

In conclusion, propolis reduces the bacterial counts inside the root canal and can be comparable with TAP as intra-canal medicament in regenerative endodontic. Moreover, both propolis and MTA as root canal orifice plugs enhance an effective induction of hard tissue deposition and soft tissue ingrowth in root canal space after revascularization of necrotic immature permanent teeth.

## Data Availability

All data used and/or analyzed during this research are available from the corresponding author on reasonable request.

## Abbreviations

CEJ: Cementoenamel junction
CFU: Colony-forming units
MTA: Mineral trioxide aggregates
NaOCl: Sodium hypochlorite
RCC: Reduction in colony counts
SD: Standard deviation
TAP: Triple antibiotic paste
